# Editorial: Insight in musculoskeletal pain—2023

**DOI:** 10.3389/fpain.2024.1411879

**Published:** 2024-04-17

**Authors:** Ashim Gupta, Laura A. Frey-Law

**Affiliations:** ^1^ Future Biologics, Lawrenceville, GA, United States; ^2^ Regenerative Orthopaedics, Noida, India; ^3^ South Texas Orthopaedic Research Institute (STORI Inc.), Laredo, TX, United States; ^4^ Department of Physical Therapy and Rehabilitative Science, University of Iowa Carver College of Medicine, Iowa City, IA, United States

**Keywords:** pain, transcutaneous electrical nerve stimulation, H-Wave® device stimulation, music, manual therapy, reactive oxygen species, COVID-19, sensory ganglia

**Editorial on the Research Topic** Insight in musculoskeletal pain—2023

Musculoskeletal (MSK) conditions are estimated to affect over 1.7 billion individuals across the globe and are one of the major reasons for chronic disability (1). MSK injuries result in pain and diminished function, and significantly affects the activities of daily living and overall quality of life of patients, leading to considerable socio-economic and healthcare burden (1). Despite its prevalence, MSK pain continues to be challenging to resolve in many cases (2). Scientific advancements are targeting this significant problem in many directions, including improved mechanistic understanding, intervention trials, and observational biomarker studies (3–7).

In our research topic, “Insight in musculoskeletal Pain—2023”, five original studies and one opinion article were published providing different perspectives on novel developments, current challenges, latest discoveries and advancements, and future perspectives on topics relevant of MSK pain. The breadth of the papers in this topic mirror the multidimensionality of the field, namely that this challenging problem will require many different perspectives to continue to make substantive advances in the field, and ultimately improve people's lived experience with MSK pain.

Two papers in the series characterized aspects of MSK pain in two different cohorts. Radojčić et al. quantified the prevalence and recurrence of MSK pain conditions using population-based surveys assessed 8 years apart in older adults in the United Kingdom (UK). MSK pain was found to be highly prevalent in older adults, with rates ranging from 20%–50%, with back and knee pain the most common. The authors identified several risk factors for MSK pain in older adults. For example, those with prior MSK pain or body mass index (BMI) values greater or equal to 27 kg/m^2^ had twice the risk of MSK. Women had higher prevalence and incidence of MSK pain, while men reported more persistent pain. This study nicely quantifies the significance of MSK pain in a cohort of older adults that are often unrecognized.

In a study characterizing a cohort of COVID-19 patients hospitalized in Iran, Erdi et al. assessed the frequency, localization and severity of pain. While dyspnea was more common (80% prevalence), pain was reported in over half of the study cohort (53.4%). Several pain manifestations were noted, including myalgia, arthralgia, headache, low back pain, bone pain, sore throat and abdominal pain, each occurring in 40%–80% of those with pain. Pain intensity was moderate with median pain visual analog scale (VAS) ratings from 4 to 6 across pain types. The results from this study demonstrate that pain can be an impactful manifestation in those hospitalized with COVID-19, which may be otherwise an unappreciated symptom.

Intervention for MSK is addressed in two papers in the series. Assel et al. prospectively evaluated physiotherapy intervention performed two times per week for 6 weeks for musicians with playing related MSK disorders (PRMD). Further, they compared characteristics between patients and matched musicians without MSK as a preliminary means to examine for risk factors for MSK. Pain improved following treatment with nearly 40% reductions observed (i.e., from 5.3 to 3.3 on average) after 12, 20-minute sessions. Higher muscle tension in several neck and upper back muscles and greater widespread pain metrics were noted in the patient group relative to the pain-free group of musicians. This study provides initial evidence supporting an individualized, conservative intervention for the treatment of musicians with playing-related MSK, a repetitive or overuse type of condition that has typically not received as much attention as work- or leisure-activity overuse syndromes., which in turn can help resume normal range of motion.

Gupta et al. in an Opinion article, compared two of the more promising forms of electrical stimulation, Transcutaneous Electrical Nerve Stimulation (TENS) and H-Wave® Device Stimulation (HWDS), and highlighted key differences between the two based on the published literature. They summarize the literature, which suggests TENS can mitigate pain, but is generally limited to small improvements (e.g., approximately 1 on a 0–10 pain scale), and consequently limited functional improvement. In contrast, they summarize the evidence suggesting HWDS has produced more sustained pain reduction (e.g., 3 point reduction on a 0–10 scale), resulting in subsequent improvement in function and reduction of pain medication use. The authors also provide a summary of several waveform parameters between the two forms of the electrical stimulation and note differences in FDA-clearances with more clinical indications approved for HWDS than TENS, despite the more widespread use of TENS.

Finally, an examination of underlying mechanisms of MSK pain, Zhang et al. examined the effect of peripheral inflammation and the accumulation of reactive oxygen species (ROS) on mechanical hyperalgesia in an animal model. The authors show accumulated ROS in the trigeminal ganglia facilitate pain hypersensitivity through activation of TRPA1, which was ameliorated with TRPA1 blockade. Further, administration of exogenous ROS into trigeminal ganglia induced mechanical hyperalgesia and spontaneous pain-like responses, further substantiating the role of peripheral inflammation and ROS in the generation of pain-like behaviors. Studies such as this which advance our understanding of underlying pain mechanisms may contribute to future efforts to develop targeted interventions.

The papers within this “Insights in Musculoskeletal Pain” series represent a few of the many perspectives converging to address this important area. We hope you enjoy this research topic!
